# Aerobic Degradation of Clothianidin to 2-Chloro-methyl Thiazole and Methyl 3-(Thiazole-yl) Methyl Guanidine Produced by *Pseudomonas stutzeri* smk

**DOI:** 10.1155/2019/4807913

**Published:** 2019-03-03

**Authors:** Satish G. Parte, Arun S. Kharat

**Affiliations:** ^1^Department of Zoology, M. J. S. College, Shrigonda, Ahmednagar, India; ^2^Department of Biotechnology, Dr. Babasaheb Ambedkar Marathwada University, Subcampus, Osmanabad, India; ^3^School of Life Sciences, Jawaharlal Nehru University, New Delhi, India

## Abstract

Overuse of pesticides in agriculture may harm environmental and agricultural yields. Sustainable maintenance of soil fertility and management of the environment have become a concern due to the persistence of pesticides in the soil. Microbes have various mechanisms for the bioremediation of persistent organic pollutants from the environment. A bacterium that degrades clothianidin was isolated from the pesticide and applied to agricultural soil by the enrichment technique. The identity of the bacterium was determined by studying morphological, cultural, and biochemical characteristics and 16S rRNA gene sequences. The ability to metabolize clothianidin was confirmed using UV-visible spectrophotometric, chromatographic, and spectroscopic analyses. A Gram-negative bacterium, designated smk, isolated from clothianidin-contaminated soil was confirmed to be a member of *Pseudomonas stutzeri*. The biodegradation of clothianidin was studied using *P. stutzeri* smk. Approximately 62% degradation of clothianidin was achieved within two weeks when grown at 30°C and pH 7. The effects of various physicochemical parameters, including pH, temperature, and clothianidin concentrations, on catabolic rates were studied. The biodegradation studies using UV-Vis spectrophotometry, HPLC, FTIR, and LC-MS indicated the production of the following metabolites: 2-chloro-5-methyl thiazole (CMT), methyl nitroguanidine (MNG), methyl 3-[thiazole-yl], and methyl guanidine (TMG). Identification of specific degradation metabolites indicates that bioremediation of toxic neonicotinoid insecticides may be achieved by application of *P. stutzeri* smk.

## 1. Introduction

Pests cause significant agronomic damage; however, damage is controlled with integrated pest management, which involves the use of different insecticides and pesticides [1]. Conversely, unplanned and extensive use of pesticides leads to an accumulation of huge amounts of residue in the environment. Uptake and accumulation of these toxic compounds in the food chain and drinking water poses serious threats to both the ecological community and human health [2].

The neonicotinoids belong to a new insecticide class, which includes the commercial products acetamiprid, nitenpyram, imidacloprid, and thiamethoxam. These products are important to agriculture because of their activity against sucking insect pests and some Heteroptera, Coleoptera, and Lepidoptera [3, 4]. Due to their broad spectrum and pesticide potential, neonicotinoid insecticides are one of the most important classes of commercial insecticides used worldwide. They are systemic, broad-spectrum insecticides, and they act as a contact or stomach poison against sucking insect pests, such as aphids, white flies, leaf- and plant-hoppers, thrips, some microlepidopteran pests, and a number of coleopteran pests [1, 2]. Their superior physicochemical properties render them useful across a wide range of application techniques, including foliar feeding, seed treatment, soil drench, and stem application [1, 5]. The high specificity of neonicotinoid compounds makes them a versatile and popular choice among all insecticides worldwide [6–10]. Among the neonicotinoids, clothianidin, ([E]-1-[2-chloro-1,3-thiazol-5-yl-methyl]-3-methyl-2-nitroguanidine), has been used broadly for the long-term control of a wide variety of insect pests, including Hemiptera, Thysanoptera, Coleoptera, Lepidoptera, and Diptera, with excellent systemic action, using a variety of application methods [11]. Clothianidin is a highly active insecticide when applied to foliage, soil, and seed and acts as an acute contact and stomach poison. They are systemic in plants and animals, are used to manage crop pests, and are also used to control pet fleas [4, 12, 13].

The extensive application of neonicotinoids has led to the introduction of extremely toxic effects on nontarget economically important insects such as honeybees and silkworms. In recent years, many problems have appeared in populations of the silkworm *Bombyx mori* (*B. mori*) as a result of pesticide applications to field crops, notably with neighboring mulberry trees [14]. The extensive application of any chemical compound eventually leads to detrimental environmental effects. The fate and disposition of neonicotinoids in the environment suggest that it is persistent, mobile, and stable to hydrolysis and has the potential to leach into ground water and flow into surface waters. In view of their toxicity and persistence, it is important that neonicotinoids be removed from the environment. Recently, the impacts of CLO, IMI, and THX on the neurological toxicity of ACE and IMI were reviewed by the European Food Safety Authority (EFSA) [15]. CLO has an undesirable environmental effect and shows unwanted toxicity to nontarget insects, such as honeybees. Therefore, EFSA and the United States Environmental Protection Agency are suspending CLO's registrations. The slow degradation of the pesticides may lead to residual amounts in the environment. For example, the accumulation of CLO in soil after multiple years of application has been reported [15, 16]. The pesticides, imidacloprid, and clothianidin are extremely persistent with a half-life of up to 3000 and 6931 days in soil, respectively [16].

Therefore, remediation of these compounds is a prime concern, and many researchers are focusing on understanding how degradation and mineralization of these compounds can be achieved by various methods, including physicochemical and biological methods [1, 17–19] (Mohammad 2005).

For the removal of such compounds from the environment, conventional methods like landfills, chemical treatment, incineration, and recycling are employed, but these methods are time-consuming and costly and lead to formation of toxic intermediates [20, 21]. Therefore, it is essential to develop efficient, eco-friendly, and economically feasible methods for pesticide detoxification. One of the most effective methods for pesticide removal is by microbial degradation by a specific degrader and/or indigenous microorganisms. The most reliable and cost-effective technique for pesticide removal is bioremediation. Among various bioremediation methods, biodegradation is the most effective technique for removal of such harmful pesticides [22]. Earlier reports have indicated that the following bacteria have the potential to degrade neonicotinoids: *Stenotrophomonas maltophilia*, *S. maltophilia* CGMCC 1.1788, *Pseudomonas* sp.1G, *Leifsonia* sp., and *Rhodotorula mucilaginosa* strain IM-2 [1, 2, 20, 21, 23, 24]. Previously, the biodegradation of clothianidin was carried out by using white rot fungus *Phanerochaete sordida* [15]. In soil, clothianidin degrades aerobically were by two main pathways. The first pathway involves the *N*-demethylation of clothianidin to form *N*-(2-chlorothiazol-5-yl-methyl)-*N*′-nitroguanidine, and *N*-methyl-*N*-nitroguanidine is formed by the cleavage of the nitroguanidine moiety in another pathway [25]. The degradation of clothianidin was also carried out by using *P. soridida*, in 2017 by Mori et al. They observed the clothianidin was transferred to TZMU and the cytochrome p450 is involved in the clothianidin degradation. Mulligan et al. [26] also reported aerobic degradation of clothianidin. Thus, we sought to isolate a clothianidin-degrading bacterium and to elucidate its degradation pathway.

## 2. Materials and Methods

### 2.1. Chemicals

Analytical grade 95% pure clothianidin was obtained from the Insecticide Residue Testing Laboratory (Pune, MS, India).

### 2.2. Isolation, Characterization, and Identification of Microorganism

A bacterial strain that is capable of clothianidin degradation was isolated from agricultural soil that received clothianidin applications for three consecutive years. For isolation of bacteria, three different variants of minimal salt medium (MSM) were used including nitrogen-limiting MSM (N-L-MSM), carbon-limiting MSM(C-L-MSM), and full-strength MSM. Enrichment techniques were used in the isolation procedures.

In total, 100 ml of MSM medium containing 0.5% g dextrose supplemented individually with 10 *μ*g/ml, 25 *μ*g/ml, or 50 *μ*g/ml of clothianidin was prepared separately in a 250 ml conical flask in duplicate. Each of the flask was inoculated with 2 g of pesticide-contaminated soil. Flasks were incubated at 30°C for seven days: one of them under static conditions and the other on a 120 rpm rotary shaker. After incubation for 7 days, microbial growth identified solitarily in the flask containing MSM with 0.5% dextrose supplemented with 10 *μ*g/ml was streak inoculated with a loop onto MSM agar plates without dextrose but supplemented with 10 *μ*g/ml of clothianidin; plates were incubated for four days at 30°C. Upon four days of incubation, colonies recovered on MSM dextrose agar supplemented with 10 *μ*g/ml clothianidin was then streaked on MSM agar supplemented with 25 *μ*g/ml clothianidin as the sole carbon source. An isolate that could grow on MSM agar supplemented with 25 *μ*g/ml clothianidin was then streaked on MSM agar supplemented 50 *μ*g/ml clothianidin as the sole carbon source. A putative clothianidin-degrading bacterial isolate that could grow at 50 *μ*g/ml clothianidin was chosen for further analysis and was stored at 4°C. The bacterial isolate was identified by conducting morphological, cultural, and biochemical tests as suggested in *Bergey's Manual of Systematic Bacteriology* followed by 16S rRNA gene sequence analysis.

### 2.3. Phylogenetic Analysis

The 16S rRNA gene sequence for *P. Stutzeri smk* was deciphered from both DNA strands. The nucleotide sequence of *P. stutzeri smk* (Acc. no. KT281608) was subjected to blast analysis using the NCBI server (http://blast.ncbi.nlm.nih.gov.in/blast.cgi). Homologous species were used in a phylogenetic analysis. The evolutionary history was inferred using the neighbor-joining method [27]. A bootstrap consensus tree that was inferred from 500 replicates was determined to represent the evolutionary history of the taxa that were analyzed [28]. The branches that corresponded to partitions that were reproduced in less than 5% of the bootstrap replicates were collapsed [29]. The evolutionary distances were computed using the maximum composite likelihood method and were expressed in units of the number of base substitutions per site [30]. The analysis included 19 high-identity nucleotide sequences. All positions containing gaps and missing data were eliminated. There were a total of 863 positions in the final dataset. Evolutionary analyses were conducted in MEGA6 [31].

### 2.4. Biodegradation Experiment

The biodegradation experiments were carried out in 100 ml of full strength MSM that contained an inoculum that had been cultured for 18 hour; clothianidin (10 mg·l^−1^) served as the single source of carbon and nitrogen. The flasks were incubated at 30°C in a rotary shaker at 120 rpm for 14 days. Bacterial growth was monitored periodically using a spectrophotometer (Hitachi-U-2800) at 530 nm. After 14 days of incubation, the flask contents were centrifuged at 10000 ×*g* for 10 min, and then, the culture supernatants were extracted using ethyl acetate in 1 : 1 (v/v) proportion. The supernatants were air-dried, were resuspended in 1 ml of HPLC grade methanol, and were used in high-pressure liquid chromatography (HPLC), Fourier-transform infrared (FTIR) spectroscopy, and liquid chromatography mass spectrometry (LC-MS) analyses. The concentrations of clothianidin were measured (250 nm) in samples obtained from the flask that was inoculated with *P. stutzeri* smk and incubated for a period of 14 days. The intrinsic loss of clothianidin was determined in samples obtained from a flask that was not inoculated with *P. stutzeri* smk.

The percent degradation of extracted compounds was calculated using the following formula: percent degradation = (Ab − Aa/Ab) 100, where Ab is the initial absorbance at 250 nm and Aa is the absorbance during the incubation period.

### 2.5. Effect of Physicochemical Parameters on Biodegradation of Clothianidin

#### 2.5.1. Effect of pH

The optimum pH for degradation was determined by cultivating bacteria in MSM supplemented with clothianidin and incubated at different pH values including pH 5, pH 7, and pH 9.

#### 2.5.2. Effect of Temperature

The optimal temperature for degradation was determined by incubating inoculated culture flasks at various temperatures including 20°C, 30°C, and 40°C.

#### 2.5.3. Effect of Initial Clothianidin Concentration on Degradation

Clothianidin at concentrations ranging from 10 g/ml to 100 g/ml was added to full-strength MSM, and the effect of the initial concentration of clothianidin on degradation was examined.

### 2.6. Analytical Studies

After 14 days of incubation, cultures were centrifuged at 10000 ×*g* for 10 min, and culture supernatant containing intermediate and terminal metabolites generated by bacterial degradation of clothianidin was extracted using ethyl acetate in a 1 : 1 (v/v) proportion. The extracted components were concentrated using a rotary evaporator and were then air-dried. The air-dried residual mass was resuspended in 1 ml of HPLC grade methanol and was further used in HPLC, FTIR, and LC-MS analyses. HPLC analysis was carried out using an isocratic system (Waters, model 2690) equipped with a C-18 column (4.6 × 250 mm) and a UV detector set at 250 nm. Methanol was used as the mobile phase at a flow rate adjusted to 1.0 ml/min.

The FTIR analysis was performed using the midinfrared (mid-IR) region of 400–4000 cm^−1^ with a scan speed of 16 (Shimadzu 8400S). LC-MS analysis of clothianidin and its metabolites was carried out using a Shimadzu LC-MS-8030 fitted with an RP-18 column (250 × 4.6 mm, 5 *μ*m). Clothianidin and its metabolites were dissolved in HPLC grade acetonitrile and were used for LC-MS analysis. The solvent system was acetonitrile-water (70 : 30, v/v) with 0.1% acetic acid at a flow rate of 0.5 ml/min. The LC-MS analysis included the following interface parameters: ESI (positive mode), nebulizing gas flow of 3 L/min, drying gas flow of 15 L/min, and DL temperature of 30°C.

#### 2.6.1. Toxicity Studies in *Mus musculus*

Five-week-old eighteen albino mice with an average weight of 20 gm were divided into three groups: 6 mice in each group. One of the three groups received subcutaneous injection of clothianidin at 1/4^th^ LD50 concentration, the second group with 0.01 mg/kg body weight of mice, while the third group with placebo (Table S1 Supplemental Information). After 24 h, incubation mice were sacrificed by the cervical dislocation method, and histopathological staining for the liver, thymus, and spleen were carried out. The studies were carried out as per the CPCSEA guidelines upon the Institutional Animal Ethical Committee approval.

#### 2.6.2. Histopathological Studies

Tissue samples were further processed for histopathological studies. They were washed in a running tap water overnight and dehydrated in ascending grades of alcohol, for which the dehydrating agent like ethyl alcohol, acetone, and isopropanol were used. Further clearing was carried out in chloroform and xylene (Supplemental information & Table S2). Following dehydration, the tissue was transferred to a paraffin solvent for infiltration. A smear of 5% Mayer's egg albumin was made onto the slide, and sections of 5 mm thickness were made with the spencer-type rotating microtome. Sections on the slide were floated in water at 55°C to 60°C. Staining was carried out upon dehydrating sections at 50°C for 30 min.

#### 2.6.3. Staining

A smear of 5% Mayer's egg albumin was prepared and smeared onto the slide and dried. With the help of the spencer-type rotating microtome, the tissue sections of 5 *μ*m thickness were taken. The tissue sections were put on the slide, and then, sections were floated in water on the slide at 55–60°C, water was drained off, and slides were dried on the hot plate at 50°C for 30 minutes. The sections were thus ready for staining (Supplemental Information and Table S3).

## 3. Results

### 3.1. Isolation, Identification, and Phylogenetic Analysis of the Isolate

**Figure 1:**
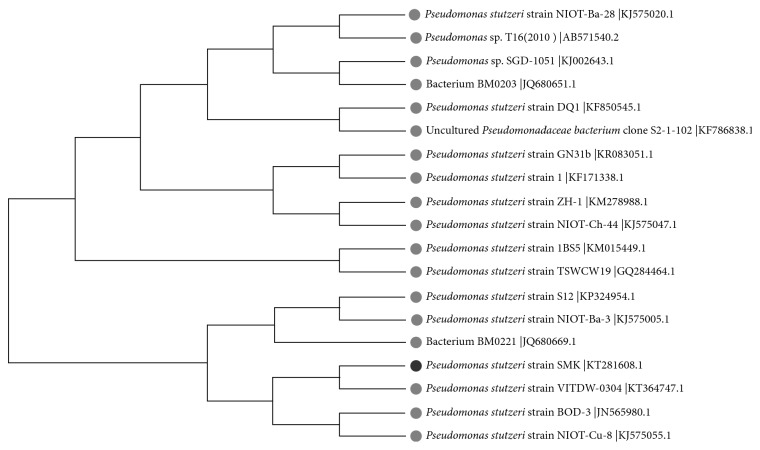
Phylogenetic analysis of the 16S rRNA gene sequence of *Pseudomonas stutzeri* smk. The dark circle indicates the isolated bacteria *Pseudomonas stutzeri* smk; the numbers above the line indicate the accession number of each strain.

A bacterium that efficiently degrades clothianidin was isolated from pesticide-contaminated agricultural soil using enrichment techniques (Materials and Methods). Bacterial identification was based on morphological and culture characteristics and biochemical tests as recommended in *Bergey's Manual of Systematic Bacteriology*. For molecular taxonomy, the 16S rRNA gene obtained from isolates was sequenced from both ends. A BLAST analysis indicated that the isolate is *Pseudomonas stutzeri*. The nucleotide sequence of the *P. stutzeri* smk16S rRNA nucleotide sequence was submitted to GenBank, KT (Accession number 281608).  Figure 1 illustrates the phylogenetic position of *P*. *stutzeri* smk compared to other species of this genus that exist in the GenBank database. The homology assay results indicated that *P. stutzeri* smk exhibits maximum similarity with the *P. stutzeri* strain VITD-0304 and the *P. stutzeri* strain BOD-3 in the phylogenetic branch.

### 3.2. Biodegradation Analysis

**Figure 2:**
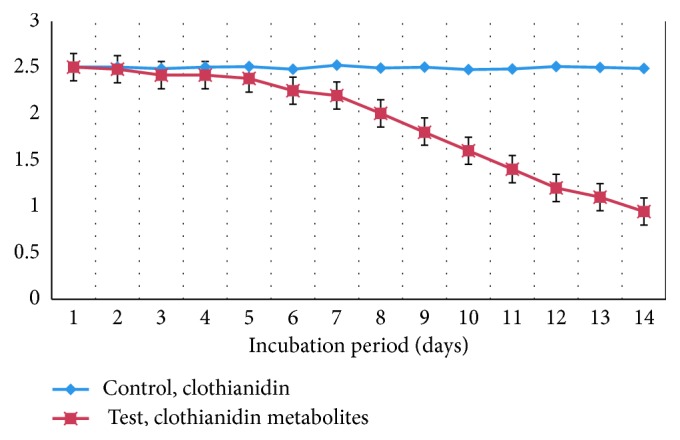
The spectroscopic analysis of clothianidin and its degradation metabolites.

Clothianidin was added to full-strength MSM, nitrogen-limiting MSM, or carbon-limiting MSM, and these solutions were used for optimization of clothianidin biodegradation. *P. stutzeri* smk showed a higher clothianidin degradation potential in full-strength MSM compared to that of nitrogen- or carbon-limiting MSM. For subsequent biodegradation experiments, we chose to use full-strength MSM. The degradation of clothianidin (as a percent) was determined spectrophotometrically.  Figure 2 shows the concentration of clothianidin measured in an extract at 250 nm following an incubation period of 14 days. *P. stutzeri* smk was capable of degrading as much as 62% of the total clothianidin within a 14-day period. It is evident from Figure 2 that the media inoculated with *P. stutzeri* smk exhibited a decline in the O.D. at 250 nm suggesting that a loss of clothianidin occurred during the 14-day period. The O.D. for the initial and final days remained approximately the same in the control flask, suggesting that there was no intrinsic loss of clothianidin during the 14-day period.

#### 3.2.1. Effect of Physicochemical Parameters (pH and Temperature) on Biodegradation of Clothianidin

**Figure 3:**
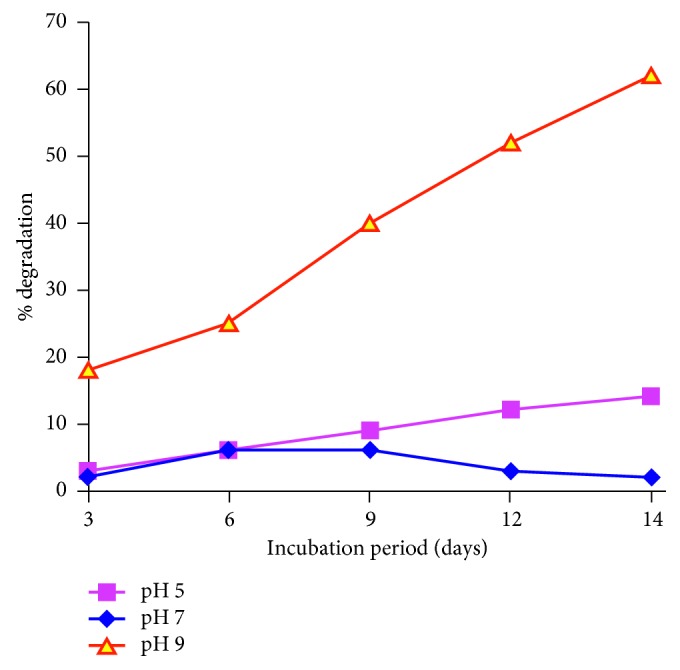
The effect of pH on the degradation of clothianidin. The graph shows the percent degradation of clothianidin at pH 5, pH 7, and pH 9.

**Figure 4:**
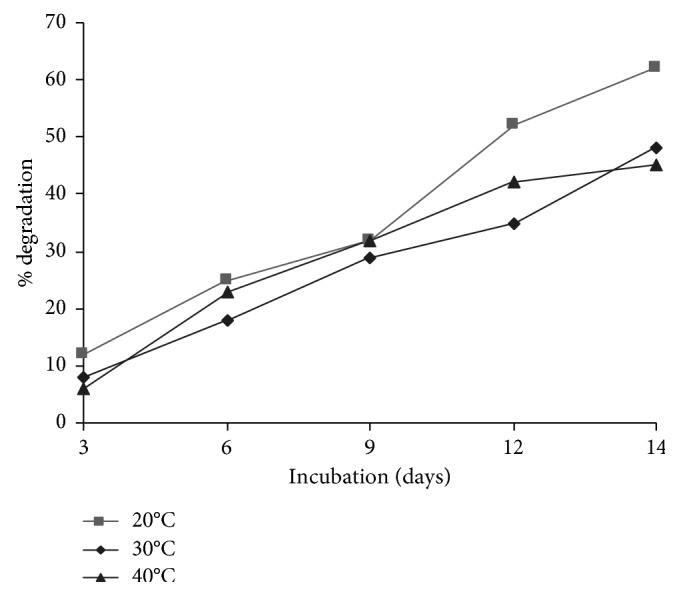
The effect of temperature on the degradation of clothianidin. The graph shows the percent degradation of clothianidin at temperatures of 20°C, 30°C, and 40°C.

 Evaluation of clothianidin biodegradation was carried out at different pH and temperature conditions. The rate of biodegradation was affected by temperature and pH. It is evident from Figure 3 that pH 7 was optimum for degradation. At pH 7, the degradation achieved within the 14-day incubation period was 62%, whereas at pH 5 and pH 9, degradation declined to 14% and 2%, respectively. Similarly, Figure 4 shows that, during the 14-day incubation period, the greatest degradation (with a maximum level of 62%) was achieved at 30°C, whereas at 20°C and 40°C, degradation gradually declined to 48% and 45%, respectively.

#### 3.2.2. Effect of Initial Clothianidin Concentration on Degradation

**Figure 5:**
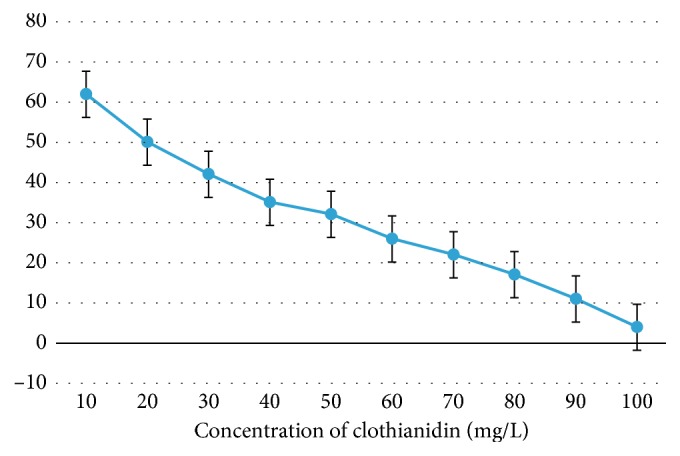
A histogram depicting the effect of the initial concentration of clothianidin on degradation. Bar 1 indicates the percent degradation of clothianidin at 10 *μ*g/l. Bars 2–10 indicate the percent degradation of dichlorovos at concentrations of 20, 30, 40, 50, 60, 70, 80, 90, and 100 *μ*g/l, respectively.

The effect of the initial clothianidin concentration on biodegradation efficacy was studied by adding clothianidin at concentrations ranging from 10 mg·l^−1^ to 100 mg·l^−1^ to full-strength MSM. The higher pesticide concentrations affected both the growth of bacteria and the rate of biodegradation. The results shown in Figure 5 demonstrate that, during the 14-day period of incubation, 62% of clothianidin degradation was achieved when grown with 10 mg·l^−1^, whereas degradation was only 4% when grown with 100 mg·l^−1^ of clothianidin. Degradation was 32% and 17% with clothianidin concentrations of 50 mg·l^−1^ and 80 mg·l^−1^, respectively.

### 3.3. Chromatographic and Spectroscopic Analysis of Clothianidin Degradation

**Figure 6:**
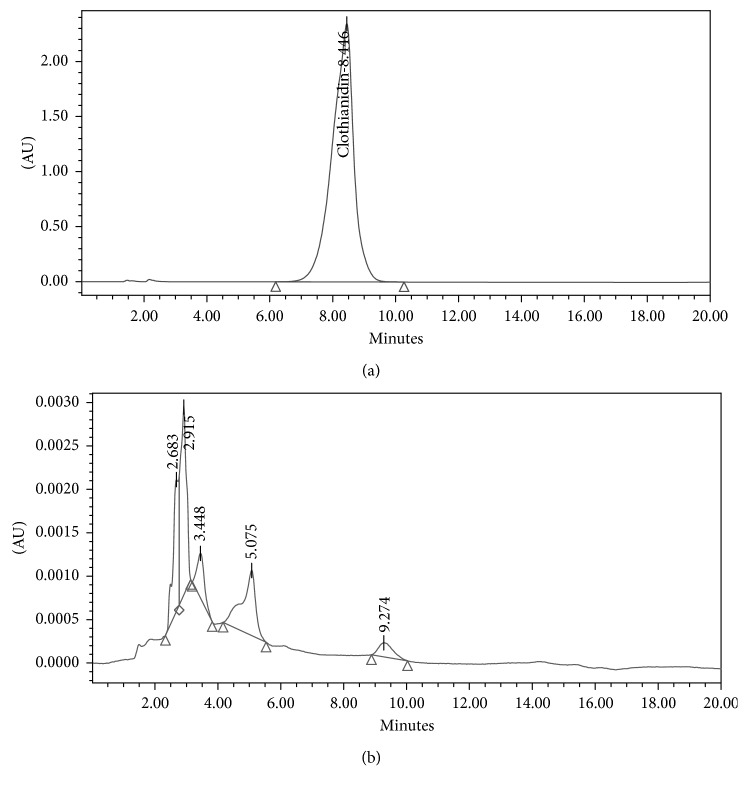
The HPLC elution profiles of a clothianidin standard and clothianidin biodegradation metabolites. The clothianidin standard shows a single peak at 8.446 RT (a); the biodegraded clothianidin shows peaks at 2.683, 2.915, 3.448, 5.075, and 9.274 RT (b).

**Figure 7:**
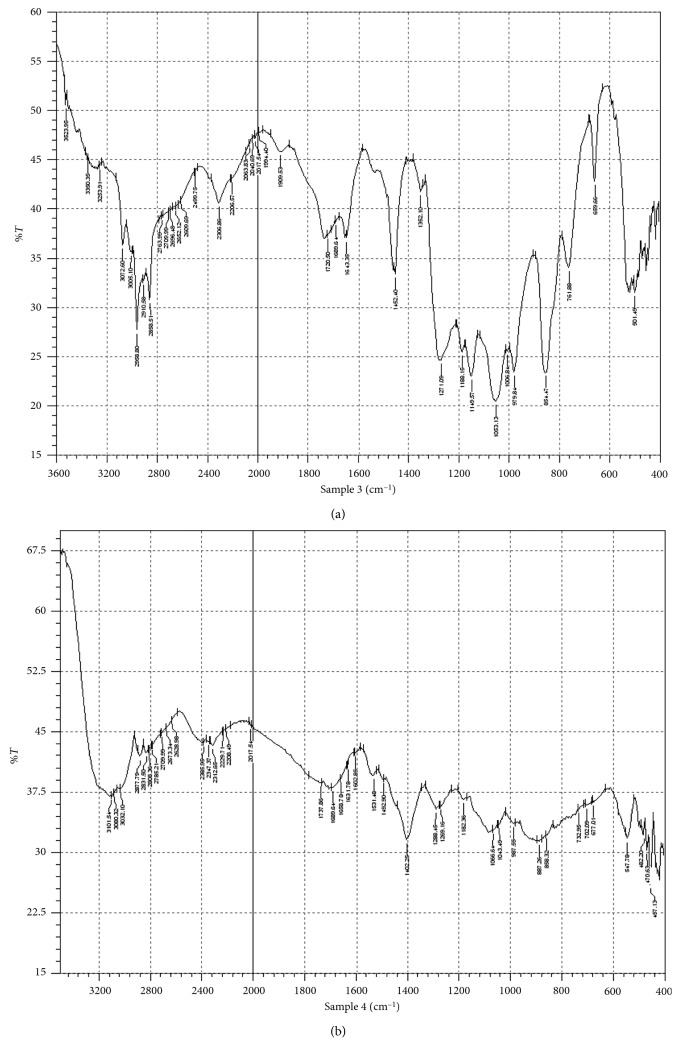
(a) The FTIR spectra of a clothianidin standard and clothianidin biodegradation metabolites. The newly formed peaks at 1737.86 cm^−1^, the broadening of peaks at 2400–3000 cm^−1^, (b) and the change in peak pattern and position indicate the conversion of clothianidin to various metabolites.

**Figure 8:**
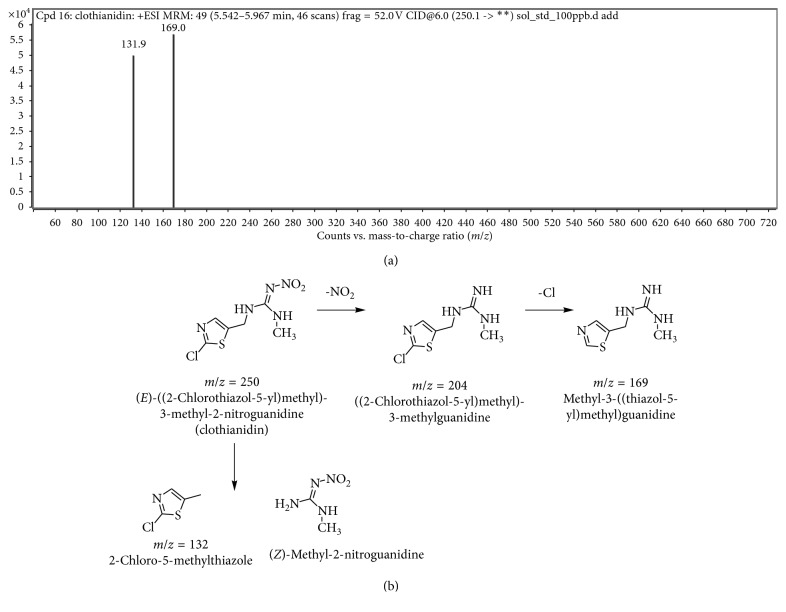
The LC-MS chromatogram of biodegradation metabolites of clothianidin (a) and the proposed degradation pathway of clothianidin (b).

The results showed that a reduced absorbance occurs at a wavelength of 250 nm, indicating that clothianidin degradation occurred. After optimizing the media and conditions in order to attain the highest possible clothianidin degradation, we sought to characterize the metabolites produced during degradation. The identification of metabolite(s) was studied with both chromatographic and spectroscopic analyses. The extracts from pure clothianidin and biodegraded clothianidin from biodegradation experiments that were carried out at optimal conditions for a period of 14 days were subjected to chromatographic comparisons of HPLC elution profiles (Methods). The results shown in Figure 6 show a single peak at a retention time of 8.446 min for pure clothianidin, whereas the degradation eluate for pure clothianidin exhibited new peaks. These new peaks exhibit an altered profile with new retention times of 2.683, 2.915, 3.448, 5.075, and 9.274 min. The overall HPLC analyses of clothianidin collectively suggest that *P. stutzeri* smk degrades clothianidin into different metabolites, and this was further substantiated by FTIR and LC-MS analyses. The FTIR analysis was performed to confirm the biodegradation of clothianidin. The results shown in Figure 7 illustrate that the FTIR spectra of pure clothianidin and degradation metabolites were considerably different from each other. The FTIR spectrum for pure clothianidin (Figure 7(a)) displayed peaks at 3331 cm^−1^ and 3250 cm^−1^ for –NH stretching, 1631.78 cm^−1^ for C=N NO2 stretching, 1541 cm^−1^ for C=N stretching in the aromatic ring, 1531 cm^−1^ and 1344 cm^−1^ for NO_2_ stretching, and 751.16 cm^−1^ for C-Cl stretching. On the contrary, the biodegradation spectra obtained with *P. stutzeri* smk (Figure 7(b)) exhibited peaks at 1737.86 cm^−1^ for C=O stretching. The change in the peak patterns and their positions within the spectra (Figures 7(a) and 7(b)) indicates that clothianidin was converted into new metabolites. The absence of peaks for N-H- stretching and NO2 stretching in spectra from extracts of pure clothianidin contrast to their presence in degraded extracts from *P. stutzeri* smk suggests that degradation results in the formation of new metabolites. The metabolites produced during clothianidin biodegradation were analyzed with LC-MS. The chromatogram of degraded clothianidin (Figure 8(a)) revealed the formation of new peaks. The mass fragmentation and *m*/*z* values were used to predict the structures of the metabolites generated during biodegradation. The clothianidin biodegradation pathway that we propose on the basis of the LC-MS analysis is depicted in Figure 8(b). The various metabolites formed during biodegradation of clothianidin include 2 chloro-5-methyl thiazole (CMT, *m*/*z* 131.9) and methyl 3-[thiazole–yl] methyl guanidine (TMG, *m*/*z*-169).

### 3.4. Effect of Degraded Metabolites on Alive Mouse

**Figure 9:**
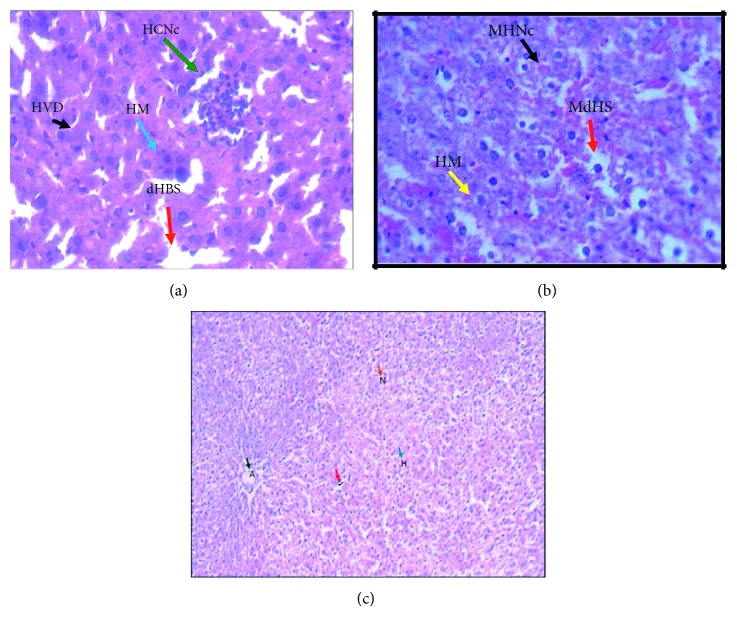
Mice were injected subcutaneously with (a) neat clothianidin and (b) degraded metabolites by *P. stutzeri* smk strain and (c) with placebo (water).

Five-week-old 18 mice were subdivided into three groups: 6 in each group. Subcutaneous injection of neat clothianidin, *P. stutzeri* smk strain-degraded metabolites, and placebo were carried out. Mice were sacrificed after 24 h, and thin sections prepared from liver tissue were strained with hematoxylin stain. Results shown in Figure 9 demonstrate that server anomalies were noticed in the liver tissue of mice challenged with clothianidin (Figure 9(a)).  Figure 9(b) demonstrate that most of these anomalies were reversed in liver tissue injected with degraded metabolites by *P. stutzeri* smk, and it is in large sense similar to that of mice challenged with sterile saline (Figure 9(c)). Histochemical analyses were also carried on the spleen (Figures S1A, S1B, and S1C) and Thymus (Figures S2A, S2B, and S2C), and results seen with these two tissues were also comparable to liver tissue, shown in Supplemental information.

## 4. Discussion

The present study was undertaken to evaluate a biological treatment and isolated bacterial culture for pesticide biodegradation. To date, very few bacterial species have been reported to have the potential to degrade clothianidin [7, 20, 21, 23]. The Gram-negative bacteria *Pseudomonas*, *Pedobacter*, and *Flavobacterium* were reported to degrade clothianidin [26]. The degradation of clothianidin was studied by Mori et al. in 2017. They observed the white-rot fungus *Phanerochaete sordida* degrades 37% of clothianidin in 20 days at 30°C [15]. In this study, we report that *Pseudomonas stutzeri* smk degrades clothianidin aerobically reaching a maximum of 62% within 14 days at 30°C. *Pseudomonas stutzeri* smk was found to degrade clothianidin faster than earlier reported for other neonicotinoid-degrading bacteria. The earlier studies used paddy field soil as inoculants for studying clothianidin degradation under aerobic and anaerobic conditions. It was clear from the earlier study that anaerobic degradation rates were significantly superior. It took more than 187 days to degrade clothianidin to 50% under aerobic conditions, whereas degradation was more advanced under anaerobic conditions: 28.3 days at 25°C and 9.3 days at 35°C [26]. Earlier studies also reported that *Stenotrophomonas* sp. THZ-XP*, Rhodotorula mucilaginosa* IM-2*, Stenotrophomonas maltophilia* CGMCC 1.1788*, Pseudomonas* sp. FH2*, Phanerochaete sordida* YK-624, *Pigmentiphaga* sp. strain AAP, and *Ochrobactrum* sp. D-12 could degrade neonicotinoid pesticides [13, 23, 32–37]. The optimization of physicochemical parameters for clothianidin biodegradation revealed that pH 7 and a temperature of 30°C are optimum for degradation. Overall, the optimization of pH and temperature is important for many microbial degradation experiments. Similar optimization studies for the biodegradation of dimethoate by *Brevundimonas* sp. MCM B-427 and of acetamiprid by *Rhodococcus* BCH 2 have been previously reported [38, 39]. Studies with increasing clothianidin concentrations revealed that the rate of biodegradation was reduced. The higher concentrations might exert a toxic effect on the cells and ultimately on the enzyme systems; therefore, higher concentrations result in decreased biodegradation. Similar results were obtained in acetamiprid degradation by *Rhodococcus* BCH 2 [39]. Furthermore, similar experiments were reported earlier in a study of the effects of the initial dye concentration in biodegradation studies of dye using various microbial systems [40]. The growth of microorganisms depends primarily on the nutrient availability in the growth medium. All of the metabolic activities of microbes are regulated through the nutrient status of the medium. When starved, microbes may switch to alternative metabolic pathways to use the available material as a food source; however, these nutrient sources may hamper the alternative metabolic activity of cells. HPLC analysis of clothianidin before and after microbial treatment confirmed the biodegradation of the pesticide into different metabolites. The results obtained from HPLC and UV-visible analyses are further supported by the results of FTIR and LC-MS analyses. LC-MS analysis was carried out to investigate the metabolites produced during the biodegradation process [41]. The chromatograms of degraded clothianidin showed the presence of two peaks. The structures of the detected compounds were assigned from the fragmentation pattern and *m*/*z* values. The pathway of clothianidin biodegradation is illustrated in Figure 8(b). It was observed that clothianidin underwent cleavage at C-N bonds between thiazolyl methyl and the guanidine moieties resulting in the formation of 2 chloro-5-methyl thiazole (*m*/*z* 131.9) and methyl nitroguanidine (*m*/*z* 118), thereby making carbon available. The present study suggests that chlorine from clothianidin does not harm bacteria. This is because the *P. stutzeri* smk prefer to use carbon and nitrogen from clothianidin by cleaving the C-N bonds between thiazolyl methyl and the guanidine moieties of clothianidin. When the carbon source is exhausted, the bacteria use the second pathway of degradation, including denitrification and dehalogenation, so that they can stepwise separate the nitrogen and chlorine resulting in the formation of methyl 3-[thiazole-5-yl]-methyl guanidine (TMG) (*m*/*z* 169). Reports generated by the registrant suggest that TZMU, TZNG, methyl-nitroguanidine, and nitroguanidine may be potential microbial transformation products under aerobic conditions [25, 42, 43]. Previously, the biodegradation of clothianidin was carried out by using white rot fungus *Phanerochaete sordida* [15]. The earlier report strongly supports the degradation of clothianidin by Gram-negative bacteria of the genus *Pseudomonas, Pedobacter*, and *Flavobacterium* (family levels Chryseobacterium, Enterobacteriaceae, and Oxalobacteraceae) [26]. In soil, clothianidin degrades aerobically by two main pathways. The first pathway involves the *N*-demethylation of clothianidin to form *N*-(2-chlorothiazol-5-ylmethyl)-*N*′-nitroguanidine, and *N*-methyl-*N*-nitroguanidine is formed by the cleavage of the nitroguanidine moiety in another pathway [25]. The degradation of clothianidin was also carried out by using *P. soridida* in 2017 by Mori et al. They observed the clothianidin was transferred to TZMU, and the cytochrome p450 is involved in the clothianidin degradation [15]. An earlier report strongly supports the hypothesis that clothianidin is degraded by Gram-negative bacteria belonging to the genera *Pseudomonas*, *Pedobacter*, and *Flavobacterium* and the Gram-positive genus *Bacillus* [26]. It was previously reported that *Pseudomonas* sp.1G converts the neonicotinoids, imidacloprid (IMI), and thiamethoxam (TMX) to their *N*-nitrosoguanidine, guanidine, and *N*-carbamoyl imine metabolites [21, 44]. Wang et al. [45] studied the toxicity of five neonicotinoid pesticides such as acetamiprid, imidacloprid, clothianidin, thiacloprid, and nitenpyram and also reported that they are highly toxic to earthworms. The insecticidal activity of neonicotinoid pesticides is based on their activity against nAChRs.

Because of the higher affinity for then AChRs of insects compared to those invertebrates neonicotinoid pesticides are broadly used in agriculture [46–48]. However, these pesticides may be neurotoxic to humans. In the biodegradation of environmental pollutants, toxicity reduction is the primary aim. Although acute and chronic toxicity tests of neonicotinoid pesticides often use insects, we evaluated the toxicity of clothianidin and their degraded intermediates by analyzing histopathological changes in the liver, spleen, and thymus of the *Mus musculus*.

The liver is the centre detoxifying any foreign compounds entering the body. So, it uniquely exposed to a wide variety of exogenous and endogenous products. These include environmental toxins and chemicals present in food or drinking [49]. Saleh in 1993, reported that the liver of treated rats with diflubenzuron, cypermethrin, and fenitrothion showed different phases of degenerative changes in the form of cloudy swelling, hydropic degeneration, chromatolysis, pyknosis, fatty degeneration, necrosis, and karyorrhexis [28]. In the same respect, data obtained by Farrag and Shalaby showed that 1/10 LD 50 of lufenuron caused venous congestion in the liver, focal necrosis of hepatocytes in the portal and periportal areas [50]. Many of the hepatocytes were pale-stained, and a few exhibited early vacuolation.

In our present study, the histopathological analysis of thymus of mice, treated with clothianidin, and the abnormalities were observed in normal architecture of the cortex and medulla. The thymic atrophy, lymphocytic depletion, invasion of fibroblasts, and focal areas of macrophage activity were observed. The similar results were observed by Mohaly et al. 2011. They reported that there was many histological changes in the thymic tissue upon treatment with imidacloprid, including lymphocytic depletion, invasion by lymphocyte, fibroblast, and occasional eosinophilic cells, pyknotic nuclei and focal areas of microphasic activity [51]. In our study, the histopathological analysis of thymus of mice treated with degraded metabolite of clothianidin showing mild abnormalities in normal architecture of cortex and medulla. The thymocyte hypertrophy and pyknotic nuclei were seen, and rare focal areas of macrophage activity were observed, suggesting the reduction in the level of toxicity upon treatment with bacteria.

The next organ of our interest was the spleen which act as the site of lymphocytic activity. Upon administration of clothianidin, the spleen of normal mice showed change in the normal architecture. The disorganization of lymphocytes in lymphoid follicles, hemosiderin deposition, depopulation, necrosis, and vascular changes was observed. Similar results were obtained by Balani et al. [52]. Mohany et al. [51] observed that the spleen of the rat treated with the IC showed the lymphocyte depletion and aggregation of pyknotic cells. Upon administration of degraded metabolites of the clothianidin, the mild alteration in the architecture as compared to the clothianidin-treated mice were observed. Moderate increase in the hemosiderin and necrosis was also observed.

The present study provides valuable information regarding the clothianidin biodegradation potential of *Pseudomonas stutzeri* smk. The strain was found to be a good candidate for faster and efficient biodegradation of clothianidin, and the study was carried out with respect to the toxicity of clothianidin and their degraded metabolites showing significant reduction in the toxicity level upon treatment of *Pseudomonas stutzeri* smk.

## Data Availability

The data used to support the findings of this study are available from the corresponding author upon request.

## Supplementary Materials

Supplementary Materials Methods: toxicity studies in *Mus musculus* five-week-old eighteen albino mice with an average weight of 20 gm were divided into three groups: 6 mice in each group. One of the three groups received subcutaneous injection of clothianidin at 1/4th LD50 concentration, the second group with 0.01 mg/kg body weight of mice, while the third group with placebo (Table S1 Supplementary Materials). After 24 h, incubation mice were sacrificed by the cervical dislocation method and histopathological staining for the liver, thymus, and spleen were carried out. The studies were carried out as per the CPCSEA guidelines upon Institutional Animal Ethical Committee approval. Housing and histopathological studies tissue sample were further processed for histopathological studies. They were washed in a running tap water overnight and dehydrated in ascending grades of alcohol, for which the dehydrating agent like ethyl alcohol, acetone, and isopropanol were used. Further clearing was carried out in chloroform and xylene (Supplementary Materials and Table S2). Following dehydration, the tissue was transferred to a paraffin solvent for infiltration. A smear of 5% Mayer's egg albumin was made onto the slide, and sections of 5 mm thickness were made with spencer type rotating microtome. Sections on the slide were floated in water at 550°C to 600°C. Staining was carried out upon dehydrating sections at 500°C for 30 min. Staining A smear of 5% Mayer's egg albumin was prepared and smeared onto the slide and dried. With the help of the spencer-type rotating microtome, the tissue sections of 5 *µ*m thickness were taken. The tissue sections were put on the slide, and then, sections were floated in water on the slide at 55–60°C, water was drained off, and slides were dried on the hot plate at 50°C for 30 minutes. The sections were thus ready for staining (Supplementary Materials and Table S3).
