# Supplementary material for: Aerobic Degradation of Clothianidin to 2-Chloro-methyl Thiazole and Methyl 3-(Thiazole-yl) Methyl Guanidine Produced by Pseudomonas stutzeri smk
Source: J Environ Public Health. 2019 Mar 3;2019:4807913. doi: 10.1155/2019/4807913 (PMC6421824; doi:10.1155/2019/4807913)
Supplement: Supplementary Materials — Methods: toxicity studies in Mus musculus five-week-old eighteen albino mice with an average weight of 20 gm were divided into three groups: 6 mice in each group. One of the three groups received subcutaneous injection of clothianidin at 1/4th LD50 concentration, the second group with 0.01 mg/kg body weight of mice, while the third group with placebo (Table S1 Supplementary Materials). After 24 h, incubation mice were sacrificed by the cervical dislocation method and histopathological staining for the liver, thymus, and spleen were carried out. The studies were carried out as per the CPCSEA guidelines upon Institutional Animal Ethical Committee approval. Housing and histopathological studies tissue sample were further processed for histopathological studies. They were washed in a running tap water overnight and dehydrated in ascending grades of alcohol, for which the dehydrating agent like ethyl alcohol, acetone, and isopropanol were used. Further clearing was carried out in chloroform and xylene (Supplementary Materials and Table S2). Following dehydration, the tissue was transferred to a paraffin solvent for infiltration. A smear of 5% Mayer's egg albumin was made onto the slide, and sections of 5 mm thickness were made with spencer type rotating microtome. Sections on the slide were floated in water at 550°C to 600°C. Staining was carried out upon dehydrating sections at 500°C for 30 min. Staining A smear of 5% Mayer's egg albumin was prepared and smeared onto the slide and dried. With the help of the spencer-type rotating microtome, the tissue sections of 5 µm thickness were taken. The tissue sections were put on the slide, and then, sections were floated in water on the slide at 55–60°C, water was drained off, and slides were dried on the hot plate at 50°C for 30 minutes. The sections were thus ready for staining (Supplementary Materials and Table S3). [file 4807913.f1.docx]

**Supplemental Information:**

**Methods:**

**Toxicity studies in *Mus musculus***

Five week old Eighteen albino mice with average weight of 20 gm were divided into three groups; 6 mice in each groups. One of the three groups received subcutaneous injection of clothianidine at 1/4^th^ LD50 concentration, second group with 0.01mg/kg body weight of mice while third group with placebo (Table S1 Supplemental Information). After 24 h incubation mice were sacrificed by cervical dislocation method, histopathological staining for liver, thymus and spleen were carried out. The studies were carried out as per the CPCSEA guidelines upon Institutional Animal Ethical Committee approval. Housing,

**Histopathological Studies**

Tissue sample were further processed for histopathological studies. They were washed in a running tap water overnight, dehydrated in ascending grades of alcohol, for which the dehydrating agent like ethyl alcohol; acetone and isopropanol were used. Further clearing was carried out in chloroform and xylene (Supplemental information & Table S2). Following dehydration, the tissue was transferred to a paraffin solvent for infiltration. A smear of 5% Mayer’s egg albumin was made onto the slide, sections of 5 mm thickness were made with spencer type rotating microtome. Sections on slide were floated in water at 55^0^C to 60^0^C. Staining was carried out upon dehydrating sections at 50^0^C for 30 min.

**Staining**

A smear of 5% Mayer’s egg albumin was prepared and smeared onto the slide and dried. With the help of spencer type rotating microtome, the tissue sections of 5µm thickness were taken. The tissue sections were put on slide and then sections were floated in water on slide at 55-60°C, water was drained off and slides were dried on hot plate at 50°C for 30 minutes. The sections were thus ready for staining (Supplemental Information and Table S3).

**Table S1**

**Treatment schedule and design:**

| Treatment | Group No. | Doses mg/kg./b.wt | Period | Dose |
| --- | --- | --- | --- | --- |
| Clothainidine | **Group I (**Normal).  **Group II.**  (Positive control).  **Group III.**  (Degraded metabolites) | 0.25 ml Water for injection  1/4 LD50 = 106.25    0.01 | 24hr. | single |

**Histopathological Studies:**

Tissue sample were further processed for histopathological studies. They were washed in a running tap water overnight, dehydrated in ascending grades of alcohol, for which the dehydrating agent like ethyl alcohol; acetone and isopropanol were used. Further clearing were carried out in chloroform and xylene. Following dehydration, the tissue was transferred to a paraffin solvent for infiltration.

**Table S2: Schedule for dehydration, clearing and paraffin infiltration:**

| **Solvent Grades** | Time |
| --- | --- |
| Alcohol 70%  Alcohol 80%  Alcohol 90%  Absolute alcohol  Isopropanol  Acetone  Chloroform  Melted paraffin wax (60 ^o^C ) | 20 min.  20 min  20 min  20 min  20 min  20 min  20 min |

The tissues were then embedded in paraffin wax to prepare tissue blocks. Tissues were then fixed in cassette after trimming them to suitable size.

**Staining method:**

**Sampling:**

After completion of the treatment period each group were sacrificed by cervical dislocation, mice were decapitated and liver, thymus and spleen were removed immediately, washed with sodium phosphate buffer (pH 7.4). Histopathological samples were fixed in 10% neutral buffered formalin and stored at 4°C for histopathological examination.

After fixing the sections on slides, the staining were carried out by serially passing them through following reagents (Table S3)

Table S3

| **Reagents** | **Time** |
| --- | --- |
| Xylol  Acetone  Alcohol  Haematoxylin stain  Running water  Eosin stain  Alcohol 95% (3 changes)  Acetone (2 changes)  Xylol (2 changes) | 3 minutes  3 minutes  3 minutes  20 minutes  20 minutes  5 minutes  3 minutes  3 minutes  3 minutes |

After passing through all the above reagents and stains, the slides were mounted with D.P.X. (Diphenyl Phthalate Xylene) and the resulting sections covered with cover slides to be ready for microscopically examinations


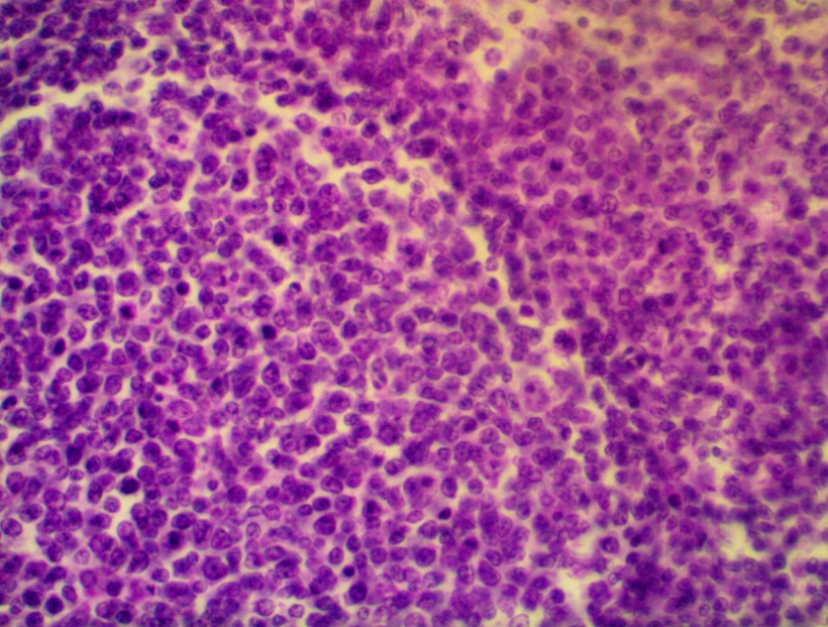


**C**

**M**

**Figure S1A: Histopathological analysis of thymus of mice (control group).showing normal architecture of cortex (C) and medulla (M). (H & E X 400).**


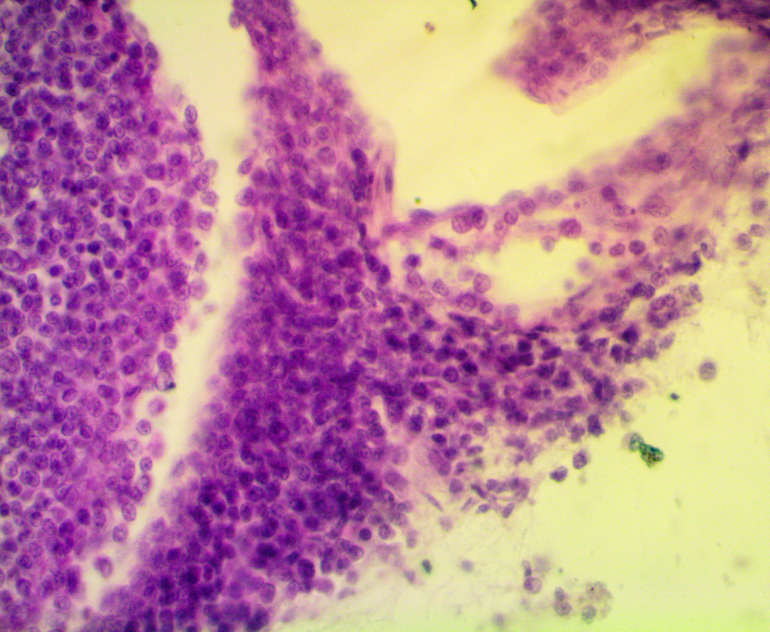


**TA**

**LD**

**MFA**

**FI**

**Figure S1B: Histopathological analysis of thymus of mice treated with clothainidine showing abnormalities in normal architecture of cortex and medulla. Thymocyte atrophy (TA), lymphocytic depletion (LD), fibroblastic invasion (FI) and focal areas of macrophage activity (MFA). (H & E X 400**).


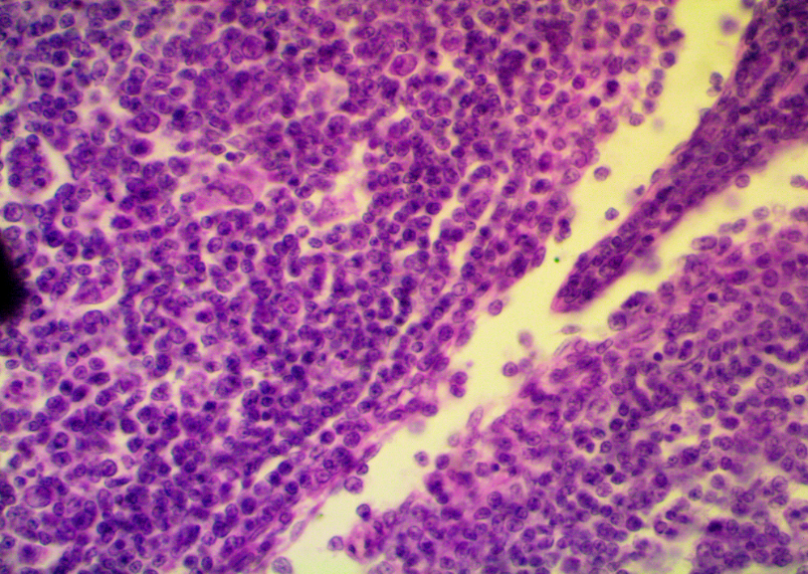


**TH**

**MLD**

**PN**

**FI**

**Figure S1C: Histopathological analysis of thymus of mice treated with degraded metabolite of clothainidine showing mild abnormalities in normal architecture of cortex and medulla. Mild lymphocytic depletion (MLD), pyknotic nuclei (PN, thymocytes hypertrophy (TH), fibroblasts infiltration (FI) and rare focal areas of macrophage activity. (H & E X 400).**


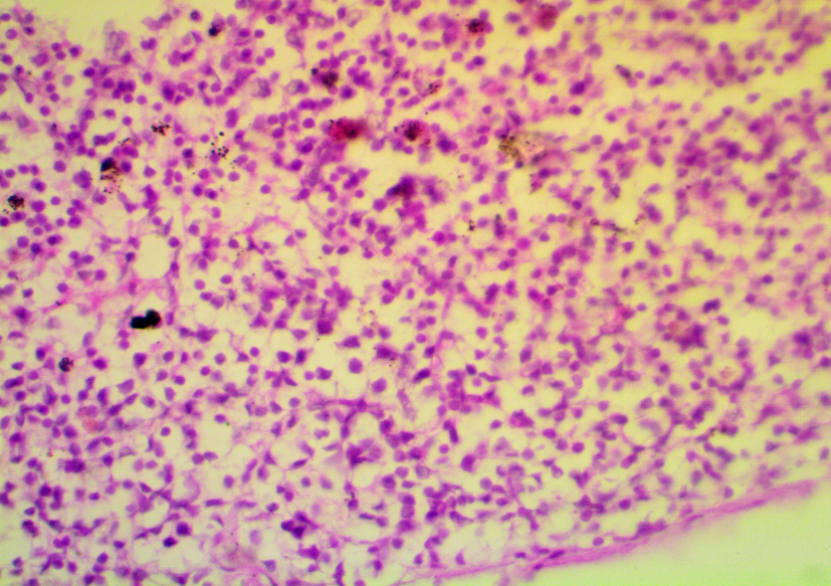


**RP**

**LN**

**LF**

**Figure S2A: Histopathological analysis of spleen of mice (control group) showing lymphatic nodules (LN) of white pulp, splenic cords of red pulp (RP) and unaltered lymphoid follicles (LF). (H & E X 400**).


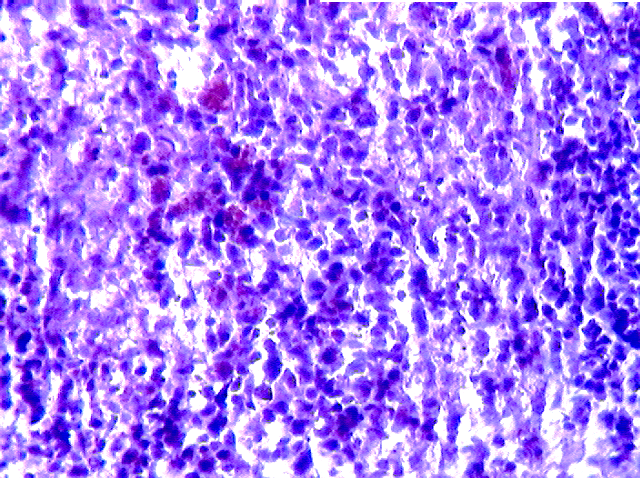


**HD**

**Nc**

**DL**

**Figure S2B: Histopathological analysis of spleen of mice (group-I) treated with clothainidine showing disorganized lymphocytes (DL) in lymphaid follicles, hemosiderin deposition (HD) and necrosis (Nc). (H & E X 400).**


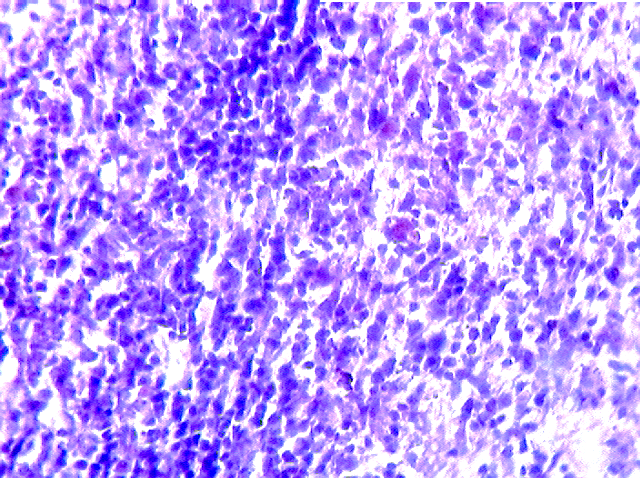


**MDL**

**HD**

**MNc**

**Figure S2C: Histopathological analysis of spleen of mice (group-II) treated with degraded metabolite of clothainidine showing moderate disorganized lymphocytes (MDL) in lymphaid follicles, few hemosiderin deposition (HD) and mild necrosis (MNc). (H & E X 400).**
